# Characterization of an antimicrobial and phytotoxic ribonuclease secreted by the fungal wheat pathogen *Zymoseptoria tritici*

**DOI:** 10.1111/nph.14786

**Published:** 2017-09-12

**Authors:** Graeme J. Kettles, Carlos Bayon, Caroline A. Sparks, Gail Canning, Kostya Kanyuka, Jason J. Rudd

**Affiliations:** ^1^ Biointeractions & Crop Protection Rothamsted Research Harpenden AL5 2JQ UK; ^2^ Plant Sciences Rothamsted Research Harpenden AL5 2JQ UK

**Keywords:** dothideomycete, host‐specific toxin (HST), microbiome, ribotoxin, *Triticum aestivum* (wheat)

## Abstract

- The fungus *Zymoseptoria tritici* is the causal agent of Septoria Tritici Blotch (STB) disease of wheat leaves. *Zymoseptoria tritici* secretes many functionally uncharacterized effector proteins during infection. Here, we characterized a secreted ribonuclease (Zt6) with an unusual biphasic expression pattern.
- Transient expression systems were used to characterize Zt6, and mutants thereof, in both host and non‐host plants. Cell‐free protein expression systems monitored the impact of Zt6 protein on functional ribosomes, and *in vitro* assays of cells treated with recombinant Zt6 determined toxicity against bacteria, yeasts and filamentous fungi.
- We demonstrated that Zt6 is a functional ribonuclease and that phytotoxicity is dependent on both the presence of a 22‐amino‐acid N‐terminal ‘loop’ region and its catalytic activity. Zt6 selectively cleaves both plant and animal rRNA species, and is toxic to wheat, tobacco, bacterial and yeast cells, but not to *Z. tritici* itself.
- Zt6 is the first *Z. tritici* effector demonstrated to have a likely dual functionality. The expression pattern of Zt6 and potent toxicity towards microorganisms suggest that, although it may contribute to the execution of wheat cell death, it is also likely to have an important secondary function in antimicrobial competition and niche protection.

## Introduction

In order to become a successful plant pathogen or parasite, a micro‐organism must not only evolve strategies to manipulate host defences and acquire nutrients, but must also interact with numerous other potential microbial competitors or antagonists present within its environmental niche. Control over these latter interactions is likely to be important for a pathogenic microbe to benefit fully from its own efforts to parasitize, and induce disease symptoms, on host plants.

For bacterial, fungal, viral and invertebrate pathogens, there exists considerable knowledge on how they manipulate their plant hosts. Studies of effectors from many different plant pathogens have revealed the important roles played by these secreted proteins in the modification of host physiology and immunocompetence (Win *et al*., 2012; Giraldo & Valent, 2013). The mechanisms by which pathogens resist host counter‐defensive measures and interact with other environmental microbes have been less fully explored by comparison. How plant‐pathogenic microorganisms interact with other microbes during infection has often been studied in the area of biological control, but the molecular interplay underpinning these often antagonistic interactions is largely unknown.

The dothideomycete fungus *Zymoseptoria tritici* is a host‐ and tissue‐specific pathogen of wheat leaves. Following spore germination on the leaf surface, there is a long phase of symptomless infection (typically at least 6–7 d), whereby the fungus invades leaves through stomata and then colonizes the mesophyll tissue (Kema *et al*., 1996). During this phase, there is minimal activation of host defences. Shortly afterwards, there is a transition to the necrotrophic phase of the life cycle. This is accompanied by massive host defence reactions, host programmed cell death, release of nutrients and the development of asexual fungal reproductive structures (pycnidia) (Kema *et al*., 1996; Keon *et al*., 2007).

The effector repertoire of *Z. tritici* has been partially characterized (Kettles & Kanyuka, 2016). Numerous bioinformatic and transcriptomic analyses have attempted to define the *Z. tritici* secretome through the infection cycle using several *Z. tritici* isolates (Mirzadi Gohari *et al*., 2015; Rudd *et al*., 2015; Palma‐Guerrero *et al*., 2016, 2017). However, to date, only a small number of candidate effector proteins have been functionally characterized. A Lysin domain effector (LysM effector), Zt3LysM (previously Mg3LysM), has been demonstrated to have chitin‐binding properties (Marshall *et al*., 2011) and is a functional orthologue of the *Cladosporium fulvum* Ecp6 effector (Bolton *et al*., 2008; de Jonge *et al*., 2010; Sánchez‐Vallet *et al*., 2013). The *Z. tritic*i *∆Mg3LysM* mutant is severely compromised in virulence (Marshall *et al*., 2011) and this effector plays a crucial role in minimizing host defence induction during early colonization through the suppression of chitin‐triggered immunity (Lee *et al*., 2014). The Necrosis and Ethylene‐inducing Peptide 1 (NEP1)‐like (NLP) family is widespread amongst plant pathogens. *Zymoseptoria tritici* produces a single NLP effector (MgNLP) (Motteram *et al*., 2009) which induces cell death in Arabidopsis, but does not induce defence genes or cell death in wheat. Recently, it has been found that numerous *Z. tritici* effectors are recognized in the non‐host tobacco species *Nicotiana benthamiana* (Kettles *et al*., 2017), and that recognition of several of these probably occurs at the plasma membrane–apoplast interface. Recently, the first avirulence gene *AvrStb6*, which also encodes a small cysteine‐rich effector, likely to be recognized by a wheat plasma membrane receptor‐like protein has been described (Zhong *et al*., 2017). However, in all cases to date, these examples of *Z. tritici* effector proteins have roles only associated with the direct or indirect manipulation of plant defences in host or non‐host plants.

In contrast with the examples above, a new concept of effector multifunctionality has recently gained traction based on data from other systems. The effector SnTox1 from the necrotrophic wheat pathogen *Parastagonospora nodorum* has recently been demonstrated to show dual functionality during wheat leaf infection (Liu *et al*., 2016). First, it induces a cell death pathway on wheat cultivars harbouring the *Snn1* sensitivity gene (Liu *et al*., 2012; Shi *et al*., 2016) and can then subsequently protect fungal hyphae from digestion by plant chitinases (Liu *et al*., 2016), presumably activated as a consequence of the wheat cell death reaction. It has been speculated that pathogen effectors may have additional roles apart from direct host interactions, specifically with regard to the manipulation of local microbial communities (microbiomes) (Rovenich *et al*., 2014). However, to date, there have been no published examples of single effector proteins with the capability to impact on the host as well as on host‐associated microbial communities.

Secreted ribonucleases (sRNases) fulfil numerous roles in many different aspects of host–pathogen interactions in both animal and plant systems. Defensive RNases are secreted by human skin cells and their inhibition is essential for protection of the producing cells (Harder & Schröder, 2002; Thomas *et al*., 2016). Two classes of sRNase of particular note are the highly toxic ribosome‐interacting proteins (RIPs) and fungal ribotoxins (Lacadena *et al*., 2007; Walsh *et al*., 2013). The toxicity of these RNases is primarily a result of their ability to cross cell membranes and to interact with ribosomes. They specifically attack the sarcin–ricin loop (SRL) of the larger eukaryotic ribosomal RNA (rRNA), irreversibly blocking protein synthesis and ultimately leading to cell death. For the fungal ribotoxins, much progress has been made to elucidate the modifications that confer potent toxicity to these proteins in comparison with non‐toxic RNases (Olombrada *et al*., 2017). In plant–pathogen interactions, it has been demonstrated recently that several RNase‐like effectors are secreted by the barley powdery mildew fungus *Blumeria graminis* f. sp. *hordei*. Although these secreted proteins are not functional RNases, they do contribute significantly to the disease progression of this biotrophic pathogen (Pedersen *et al*., 2012; Pliego *et al*., 2013).

All studies into the role of *Z. tritici* effectors to date have focused on their function in wheat colonization or potential recognition in non‐host plants, whereas the potential for multifunctional effectors with roles in microbe–microbe interactions has not yet been explored. Here, we describe an sRNase (Zt6) from *Z. tritici* which is expressed specifically during distinct phases of wheat leaf infection, and which possesses highly potent cytotoxic activity against plants as well as various prokaryotic and eukaryotic microbes. This activity appears to be reliant on an N‐terminal region of the protein which may facilitate cellular uptake into target cells. Conversely, Zt6 was found to be non‐toxic to *Z. tritici* itself, suggesting the existence of an unknown self‐protection mechanism.

## Materials and Methods

### Plant growth conditions

Seeds of *Nicotiana benthamiana* (tobacco) were sown and transplanted as described previously (Kettles *et al*., 2017) and maintained in a glasshouse at 16 h 22°C : 8 h 18°C, day : night. Seeds of *Triticum aestivum* (wheat) cultivar (cv) Riband were sown directly in half‐trays of Rothamsted Prescription Mix and maintained in a glasshouse at a constant 17°C with a 16 h day length.

### Fungal growth and inoculations

All *Z. tritici* strains were grown on yeast extract–peptone–dextrose (YPD) agar plates at 16°C for 5 d. Conidiospores were harvested in distilled H_2_O containing 0.01% Tween‐20 and plant inoculations were performed as described previously (Kettles *et al*., 2017).

### *Agrobacterium*‐mediated targeted deletion of *Zt6*

*Agrobacterium tumefaciens*‐mediated transformation of the *Z. tritici* IPO323 *∆ku70* strain (Bowler *et al*., 2010) was performed as described previously (Motteram *et al*., 2009). A construct designed to replace the *Zt6* (*Mycgr3G38105*) gene with the hygromycin resistance gene was produced using the vector pCHYG and validated by diagnostic PCR. All primers used in the production of the gene deletion construct and in sequence validation are available in Supporting Information Table S1.

### *Agrobacterium*‐mediated transient expression of *Zt6* in *N. benthamiana*

Gateway cloning of *Zt6* into the binary vectors pEAQ‐HT‐DEST3 and pEARLEYGATE101 was conducted as described for other *Z. tritici* candidate effectors (Kettles *et al*., 2017).

### Phylogenetic analysis

Mature Zt6 amino acid sequence (lacking secretion signal peptide (SP)) was used as input for blastp analysis against all Dothideomycete sequences at NCBI (National Center for Biotechnology Information). The 20 closest homologues (Table S2) were aligned to Zt6 using ClustalW, and a phylogenetic tree was constructed using the maximum likelihood (ML) method with 100 bootstrap replicates in Mega6. Amino acid sequence representations of the predicted N‐terminal ‘loop’ and downstream ribonuclease (RNase) domains were produced using weblogo3 (http://weblogo.threeplusone.com) employing the same ClustalW alignment as input.

### Site‐directed mutagenesis

Generation of the *Zt6‐H70A* mutant was performed using the QuikChange Lightning Multi Site Mutagenesis Kit (Agilent Technologies, Santa Clara, CA, USA) following the manufacturer's instructions. Primers were designed using the QuikChange Primer Design program and are included in Table S1.

### Quantitative reverse transcription‐polymerase chain reaction (qRT‐PCR)

Expression analysis of Zt6 was performed using sample material generated from *Z. tritici* IPO323 grown in YPD medium and at 1 d post‐inoculation (dpi) of susceptible wheat cv Riband. Total RNA was recovered using Trizol (Thermo Fisher Scientific, Waltham, MA, USA) with a double chloroform extraction step. RNA preparations were treated with RQ1 DNase (Promega) following the manufacturer's instructions. DNA‐free RNA was recovered by ethanol precipitation, and 2 μg of RNA was used as template for cDNA synthesis employing SuperScript III reverse transcriptase (Thermo Fisher Scientific) and an oligo(dT)_20_ primer. cDNA was diluted 1 : 2 with distilled H_2_O and 1 μl of diluted cDNA was used in each reaction with SYBR Green Jumpstart Taq ReadyMix (Sigma). Triplicate reactions were prepared for each sample–primer pair combination and all reactions were performed using the following thermocycle on a Bio‐Rad CFX384 real‐time system (3 min at 95°C, followed by 40 cycles of 30 s at 95°C, 30 s at 60°C, 30 s at 72°C, followed by melt curve analysis of 10 s at 95°C, then 65–95°C in 0.5°C increments, 5 s at each). Three biological replicate samples were included for each treatment. Relative expression values were calculated using the 2^−∆Ct^ method with *Z. tritici beta‐tubulin* as the reference gene. Expression values were rescaled for presentation such that the YPD treatment for each effector is equal to 1. All primer sequences are provided in Table S1.

### Wheat leaf sheath biolistic bombardment

*Zt6* variants were cloned into the pRRes14_RR.1m201_125 biolistic bombardment vector linearized by double digestion with *Nco*I and *Spe*I restriction endonucleases. Corresponding *Nco*I/*Spe*I recognition sites were added to *Zt6* fragments by PCR amplification, followed by double digestion with the corresponding restriction endonucleases. *Zt6* fragments were incorporated into linearized pRRes14_RR.1m201_125 using T4 DNA ligase and transformed into One Shot TOP10 electrocompetent *Escherichia coli* (Thermo Fisher Scientific) using standard procedures. Constructs were sequence verified by Sanger sequencing. All primer sequences used are available in Table S1.

For bombardments, young tillers were collected from *c*. 4‐wk‐old wheat cv Apogee plants. A section *c*. 10 cm in length containing the immature inflorescence was cut from each tiller and the ends were sealed with parafilm. The trimmed stems were surface sterilized using 70% v/v ethanol for 3 min and 10% v/v domestic thin bleach (sodium hypochlorite content of 4–6%) for 3 min, followed by several repeat washes with sterile water. Leaf sheaths were isolated by cutting away the outer layers to expose the immature inflorescence, and *c*. 1–1.5 cm of the young leaf sheath closest to the inflorescence was removed. The isolated leaf sheaths were plated on L7 medium supplemented with 3% w/v sucrose, 0.5 mg l^−1^ 2,4‐dichlorophenoxyacetic acid (2,4‐D) (Sigma‐Aldrich, UK) and 10 mg l^−1^ AgNO_3_ (Sigma‐Aldrich, UK), and solidified with 5 g l^−1^ agargel (Sigma‐Aldrich, UK) in 9‐cm Petri dishes, placing sufficient leaf sheath sections to cover the central 2 cm^2^. The prepared leaf sheaths were transformed on the same day as isolation. Particle bombardment was carried out according to Sparks & Jones (2014). Test constructs were precipitated onto 0.6‐μm gold particles (Bio‐Rad, UK) alongside a construct which contained the green fluorescent protein (GFP) reporter gene under the control of the maize ubiquitin gene promoter with an *Arabidopsis thaliana* histone H2B‐like nuclear targeting sequence and nopaline synthase (*nos*) gene terminator. The particles were delivered into target tissues using a Bio‐Rad PDS‐1000/He™ particle gun with a rupture pressure of 650 psi and a vacuum of 28–29 inches Hg. Treated leaf sections were incubated at *c*. 22°C in the light (12 h photoperiod) for 2 d before visualization of GFP using a Leica M205 FA stereomicroscope (Leica Microsystems, Wetzlar, Germany) with a fluorescence filter suitable for GFP.

### RNase activity assays

Wild‐type (FL) and mutant *Zt6* (∆19–40, H70A, ∆19–40 + H70A) were recombined into the Gateway‐compatible pDEST17 expression vector (Invitrogen) using LR clonase II enzyme mix following the manufacturer's instructions. Sequence‐verified constructs were linearized by digestion with *Eco*RV restriction endonuclease and 1 μg of linearized plasmid was employed as template for *in vitro* transcription reactions (carried out for 3 h at 37°C) using the MEGAscript T7 transcription kit (Ambion). Transcription products were purified using the RNA cleanup protocol of the RNeasy Mini Kit (Qiagen). *In vitro* translation reactions were assembled using the Rabbit Reticulocyte Lysate/Wheat Germ Extract Combination System (Promega) employing 1 μg RNA as template. The provided luciferase control RNA was used as negative control for RNase activity. Reactions were incubated for 10–90 min at 30°C (rabbit reticulocyte lysate) or 25°C (wheat germ extract). Ribosomal RNA was recovered following the method of Kao *et al*. (2001) before resolving on 2% semi‐denaturing agarose gels.

### *Tobacco rattle virus* (TRV)‐mediated virus‐induced gene silencing (VIGS) in *N. benthamiana*

This method has been fully described previously (Kettles *et al*., 2017). Briefly, 2–3‐wk‐old *N. benthamiana* seedlings were agroinfiltrated with a 1 : 1 mix of *Agrobacterium* strains harbouring PTV00 (TRV RNA2)‐derived constructs and *Agrobacterium* GV3101 carrying pBINTRA6 (TRV RNA1) at a final optical density at 600 nm (OD_600_) = 1 to initiate silencing. After 2–3 wk, full‐length Zt6 was expressed in systemically infected leaves by agroinfiltration of GV3101, carrying pEAQ‐HT‐DEST3 Zt6, at OD_600_ = 1.2. Leaves were visually assessed for cell death symptoms at 7 dpi.

### Recombinant protein expression and purification

*Zt6* variants were cloned into the p75 protein expression vector (Franco‐Orozco *et al*., 2017) linearized by digestion with *Pac*I using yeast recombination cloning in *Saccharomyces cerevisiae* FY834. Recombined plasmid DNA (p75 Zt6 ∆19–40 or p75 Zt6 H70A) was recovered from *S. cerevisiae* and bulked up by transformation into One Shot TOP10 electrocompetent *E. coli* (Thermo Fisher Scientific). Plasmids were sequence verified by Sanger sequencing and constructs were linearized by digestion with *Pme*I before electrotransformation into *Pichia pastoris* GS115 cells. *P. pastoris* (p75 Zt6 ∆19–40 or p75 Zt6 H70A) transformants were selected by growth on YPD supplemented with zeocin (100 μg ml^−1^) for 2–3 d at 30°C. For the identification of colonies highly expressing recombinant proteins, 2 ml of YPD cultures were prepared for 10–15 individual colonies of each construct and grown for 48 h at 30°C (250 rpm) in a shaker incubator. Cells were pelleted by centrifugation (2000 ***g*** for 5 min) and 2 μl of supernatant was spotted onto nitrocellulose membrane (Amersham Protran Premium 0.45 NC; GE Healthcare, Chicago, IL, USA). Membranes were incubated in blocking buffer (TBST + 2% milk powder) and then probed with anti‐V5 horseradish peroxidase (HRP)‐conjugated monoclonal antibody (E10/V4RR; Pierce, Waltham, MA, USA) at 1 : 5000 dilution in blocking buffer before washing in TBST. Signal was detected using an Amersham ECL prime kit (GE Healthcare) and exposure to X‐ray film. For scaled‐up purification, transformants highly expressing recombinant proteins were grown in flasks containing 1.5 l YPD supplemented with zeocin (50 μg ml^−1^) for 48 h at 30°C (250 rpm) in a shaker incubator. Cells were pelleted by centrifugation, and supernatant was incubated with anti‐V5 affinity gel (Biotool/Stratech Scientific Ltd, Newmarket, UK) following the manufacturer's instructions. Affinity gel was washed thrice in TBS and protein was recovered by 5 × 1 ml elutions with elution buffer (0.25 × TBS, 200 μg ml^−1^ V5 peptide). Protein elutions were pooled, filter sterilized and buffer exchanged to distilled H_2_O using Amicon Ultra‐15 columns (3 kDa MWCO, Merck Millipore), such that the final concentration of V5 peptide was ≤ 1 μg ml^−1^. Purified proteins were quantified by spectrophotometry measuring the absorbance at 280 nm (A_280_).

### Microbial toxicity assays

Lysogeny broth (LB) or YPD liquid cultures with appropriate antibiotics were inoculated with *E. coli* Top10 (pEAQ‐HT‐DEST3 Avr4), *P. pastoris* GS115 (p75 EV) or *S. cerevisiae* FY834 and grown overnight at 30°C. Cells were pelleted by centrifugation at 2000 ***g*** (JA20 rotor) for 5 min, washed once in distilled H_2_O and recovered as above. *E. coli* cells were diluted with distilled H_2_O to a final OD_600_ = 0.01, with *P. pastoris* and *S. cerevisiae* diluted to OD_600_ = 0.6. The *Z. tritici* IPO323 *∆ku70* strain was grown for 5 d at 16°C on YPD agar plates supplemented with geneticin. Conidiospores were resuspended to a density of 1 × 10^7^ spores ml^−1^ in distilled H_2_O + 0.01% Tween‐20. Reaction mixes were prepared by combining 10 μl cells at the stated OD_600_ or spore density with recombinant Zt6, restrictocin (Sigma) or RNaseA (Qiagen) at 20 μM final concentration, with distilled H_2_O added to a final reaction volume of 30 μl. Reactions were incubated for 24 h with gentle agitation. Serial dilutions of 10‐μl aliquots of each reaction were prepared and plated on appropriate selection media and grown at 30°C (*S. cerevisiae*,* P. pastoris*,* E. coli*) or 16°C (*Z. tritici*) until colonies appeared.

## Results

### A *Z. tritici* gene *Zt6*, encoding a small sRNase, shows biphasic upregulation during infection of susceptible wheat

**Figure 1:**
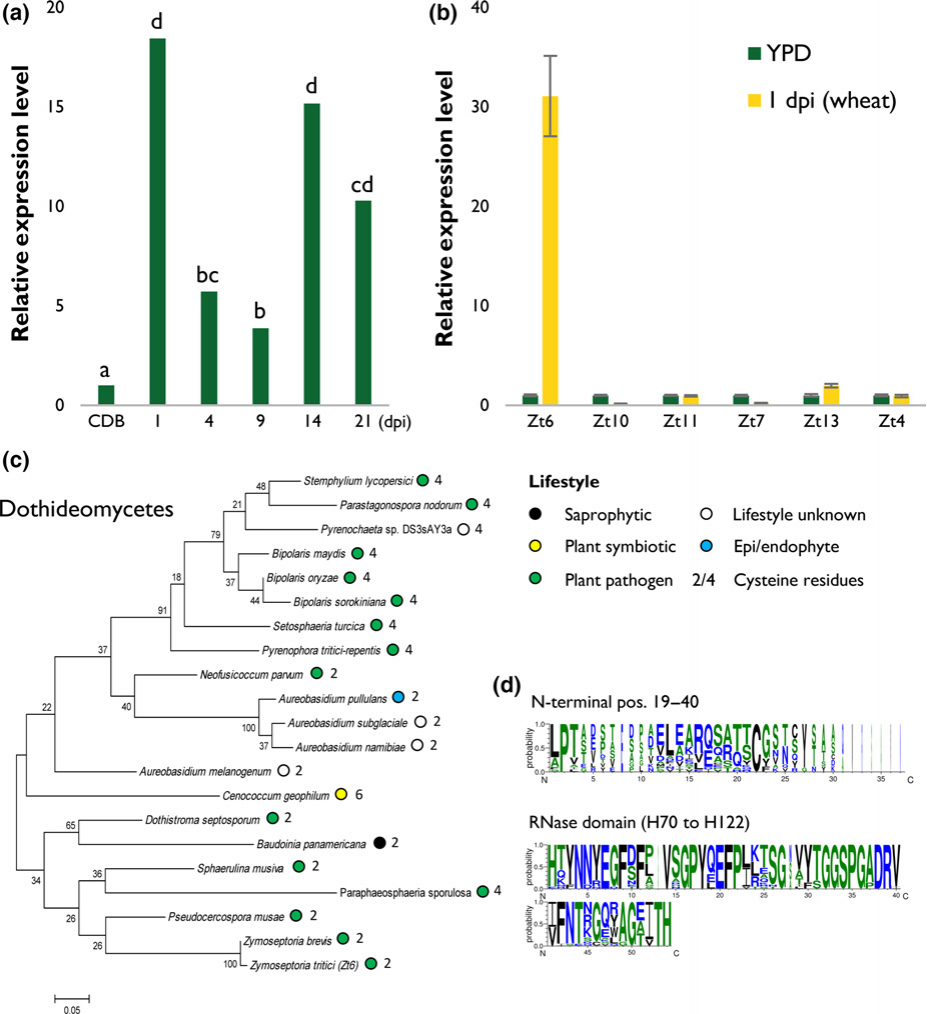
Transcriptomic and phylogenetic analysis of *Zymoseptoria tritici* effector Zt6. (a) RNAseq expression profile of *Zt6* (*Mycgr3G38105*) from *Z. tritici* IPO323 infection time course (1–21 d post‐inoculation, dpi) of susceptible wheat (*Triticum aestivum*) cv Riband in comparison with growth in Czapek–Dox broth (CDB) (Rudd *et al*., 2015). Letters indicate significant differences at *q* < 0.05. (b) Quantitative reverse transcription‐polymerase chain reaction (qRT‐PCR) expression profile of *Zt6* from *Z. tritici* IPO323 grown in yeast extract–peptone–dextrose (YPD) broth in comparison with 1 dpi growth on susceptible wheat cv Riband. Bars represent mean ± SE. (c) Phylogenetic representation of the closest 20 Zt6 orthologues within the Dothideomycota. Coloured circles represent the lifestyle of each species in which orthologues are present. Sequences were obtained by blastp (NCBI) and aligned using ClustalW. The tree was constructed using the maximum likelihood (ML) method with 100 bootstrap replicates (mega6). (d) WebLogo representations of the 22‐amino‐acid N‐terminal (residues 19–40) and ribonuclease (RNase) core (residues 70–122) domains of Zt6 and the 20 closest Dothideomycete orthologues.

A recent transcriptomic analysis of *Z. tritici* isolate IPO323 infection of the susceptible wheat cv Riband (Rudd *et al*., 2015) revealed *in planta* expression of an sRNase (*Mycgr3G38105*, hereafter referred to as *Zt6*) of the N1/T1 class. Expression at all time points during wheat infection was significantly higher than during growth in Czapek–Dox broth (CDB) (Fig. 1a; Table S3). *Zt6* exhibited a double‐peak expression pattern during wheat infection, with maximal expression at 1 dpi and a secondary peak at 14 dpi (Fig. 1a; Table S3). These time points coincide with spore germination immediately following leaf surface inoculation (1 dpi) and the necrotrophic phase of the fungal life cycle (14 dpi), respectively. The expression of *Zt6* and several other genes encoding effector proteins (Kettles *et al*., 2017) in an *in vitro* culture of *Z. tritici* IPO323 grown in nutrient‐rich (YPD) medium vs *in planta* at 1 dpi on susceptible wheat cv Riband was also analysed by qRT‐PCR (Fig. 1b). In agreement with our previous data (Rudd *et al*., 2015), *Zt6* expression was considerably higher at 1 dpi during wheat infection relative to *in vitro* growth in YPD culture. Furthermore, the induction of *Zt6* was more rapid than the induction of several other *Z. tritici* candidate effectors tested (Fig. 1b). Collectively, these data indicate that *Zt6* is rapidly induced following spore germination on the wheat leaf surface in comparison with growth *in vitro*.

### *Zt6* is widely conserved within the Dothideomycetes

*Zt6* encodes a protein of 137 amino acids, with residues 1–18 corresponding to a secretion signal peptide (SP) predicted using SignalP and TargetP. The predicted RNase domain represents most of the mature protein sequence and is characterized by a histidine–glutamic acid–histidine (HEH) catalytic triad at amino acid positions 70, 88 and 122, respectively. Blastp analysis using the mature Zt6 protein sequence identified no paralogous genes in *Z. tritici* and revealed that homologues (between one and three per species) are present in many Ascomycete fungi, and in all species with sequenced genomes within the class Dothideomycetes (Fig. 1c). The closest homologues were found in species with a pathogenic lifestyle, although singular examples were also found in fungi described as epi/endophytic, saprophytic or symbiotic. Mature Zt6 protein (residues 19–137) contains two cysteine residues (residues 36 and 133) which are not predicted to form a disulfide bridge (DiANNA, SCRATCH protein predictor DIpro). This two‐cysteine form of sRNase is also found in the majority of most closely related plant‐pathogenic fungal species, including *Zymoseptoria brevis* and *Pseudocercospora musae*, which, like *Z. tritici*, are also members of the *Mycosphaerellaceae* (Fig. 1c). However, many pathogens, including important wheat leaf‐infecting species, such as *P. nodorum*,* Bipolaris sorokiniana* and *Pyrenophora tritici‐repentis*, encode a four‐cysteine form which may impact protein folding. Analysis of the overall sequence diversity between Zt6 homologues indicated that most variation is seen at the N‐terminal end of the mature protein (residues 19–40 shown), whereas there is a high level of conservation within the RNase functional domain (H70–H122 shown) (Fig. 1d).

### Zt6 encodes a functional cytotoxic protein active against wheat leaf cells

**Figure 2:**
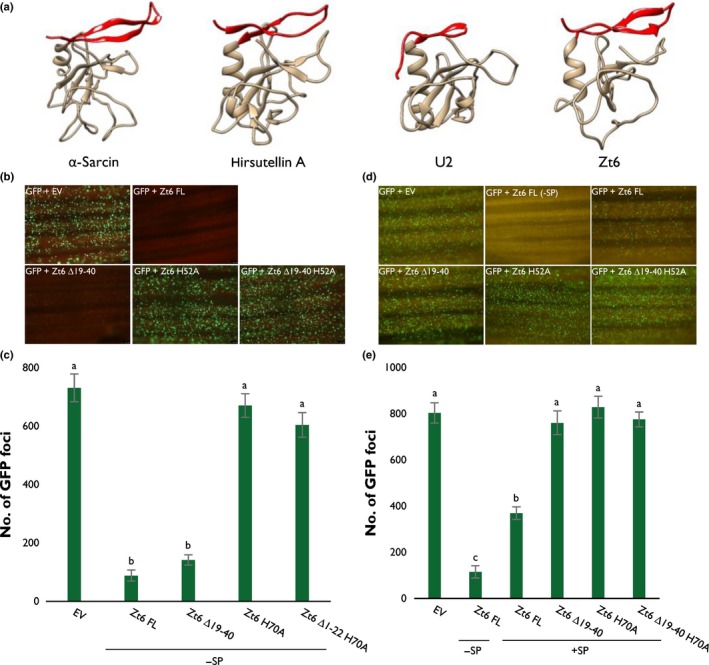
Zt6 inhibits transgene (green fluorescent protein, GFP) expression in wheat (*Triticum aestivum*) leaves. (a) Protein folding prediction using Phyre2 for Zt6 alongside known structures of two ribotoxic ribonucleases (RNases) (α‐sarcin, hirsutellin A) and the non‐cytotoxic RNase U2. The N‐terminal loops (residues 19–40 for Zt6) are highlighted in red. The images were produced using the Chimera Molecular Modeling System (University of California, San Francisco, CA, USA, http://www.rbvi.ucsf.edu/chimera). (b–e) Biolistic co‐bombardment of GFP‐ and Zt6‐expressing constructs (full‐length (FL), N‐terminal loop mutant (∆19–40), catalytic mutant (H70A), and double mutant (∆19–40 + H70A)) into wheat cv Apogee leaves. Zt6 variants were expressed both without signal peptide (− SP) (b, c) and with signal peptide (+ SP) (d, e). All leaves were photographed at 2 d post‐inoculation (dpi) and GFP foci were counted using Metamorph software (Molecular Devices, Sunnyvale, CA, USA). Bars represent mean ± SE. Letters indicate differences at *P *<* *0.001 as determined by *t*‐probabilities within a generalized linear model (GLM).

To assess potential Zt6 toxicity in the natural host (wheat), we used biolistic bombardment to coexpress a GFP transgene alongside mature Zt6 (–SP) (Fig. 2b,c) within host epidermal cells. In comparison with the GFP + empty vector (EV) control treatment, GFP + Zt6 (− SP) almost completely abolished GFP fluorescence, thus indicating potent cellular toxicity when expressed in wheat leaf cell cytoplasm. These data also established that the wheat bombardment assay was a suitable and high‐throughput system for the further characterization of the Zt6 protein activity.

### The N‐terminal loop region of the mature Zt6 sRNase likely functions in enabling protein re‐entry into wheat cells

It is known that the N‐terminal region of secreted RNases can play an important role in cellular toxicity. The ribotoxin class of RNases, for example, possesses elongated N‐terminal loops in a β‐sheet–hinge–β‐sheet structure that have been demonstrated to play a role in ribosome binding and have been postulated to be involved in cellular uptake (Garcia‐Ortega *et al*., 2002; García‐Mayoral *et al*., 2005), both important features that contribute to the cytotoxicity of these molecules. By contrast, non‐toxic RNases have comparatively short N‐terminal loops (Lacadena *et al*., 2007). A structural prediction for Zt6 using Phyre2 (Kelley *et al*., 2015) suggests that an N‐terminal loop region exists with similarity to the atypical ribotoxin hirsutellin A from the mite fungal pathogen *Hirsutella thompsonii* (Herrero‐Galán *et al*., 2008) (Fig. 2a). We speculate that this region may be important for Zt6 function and that Zt6 may exhibit some characteristics of a ribotoxic RNase.

To investigate the contribution of the N‐terminal loop to cytotoxicity, a *Zt6 ∆19*–*40* mutant missing the first 22 amino acids of the mature protein (Fig. 2a, shown in red) was coexpressed with GFP in the wheat biolistic system. This resulted in a near‐complete absence of GFP fluorescence, indicating that this truncation mutant retains near‐wild‐type activity when expressed intracellularly (i.e. without the secretion signal sequence). By contrast, coexpression of an RNase catalytic mutant (H70A) or the double mutant (∆19–40, H70A) permitted GFP fluorescence at levels similar to those of the EV control (Fig. 2b,c), indicating a near‐complete absence of toxicity. Together, this indicates that RNase activity is required for Zt6 toxicity, but that an N‐terminal loop is dispensable for this activity when the protein is retained (or expressed) intracellularly.

To test whether the N‐terminal loop region might instead be important for protein uptake into wheat cells, a complementary set of bombardments was conducted with both mature Zt6 protein and the mutants described above, with a re‐introduced secretion signal to direct the Zt6 protein to the apoplastic space of wheat cells, and its effects on intracellular GFP fluorescence were monitored (Fig. 2d,e). Full‐length Zt6 (containing its secretion signal) was still able to strongly inhibit GFP fluorescence during co‐bombardment, suggesting that this secreted fungal protein can re‐enter the host cell cytoplasm, where presumably it exerts its cytotoxic activity. Interestingly, and in stark contrast, Zt6 ∆19–40 expressed with the secretion signal induced no or very little host cell death as GFP expression was not compromised. Indeed, the activity of the ∆19–40 mutant was approximately equivalent to the EV control and the Zt6 H70A catalytic mutant (Fig. 2d,e). These data indicate that the N‐terminal 22 amino acids of mature Zt6 are important for toxicity against wheat cells only when the protein is initially directed to the apoplastic space of wheat leaves. The most likely explanation for this effect is that the N‐terminal loop may be required for Zt6 re‐entry into host cells.

### Both mature Zt6 and the ∆19–40 mutant protein are fully functional RNases active against rRNA

**Figure 3:**
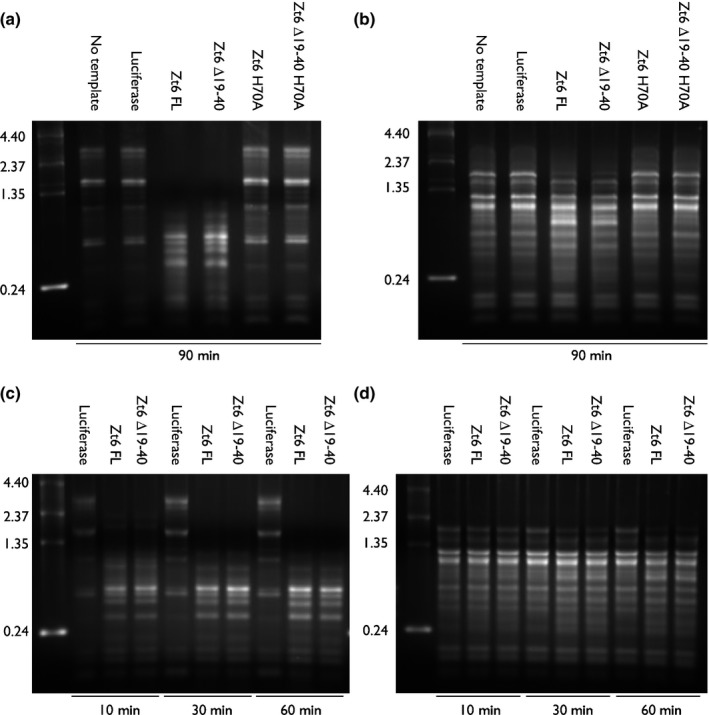
Zt6 is a functional ribonuclease (RNase) with catalytic activity against native rRNA from animal and plant ribosomes. Gel electrophoresis of recovered rRNA from (a, c) rabbit reticulocyte lysate and (b, d) wheat germ (*Triticum aestivum*) extract producing recombinant Zt6 FL, Zt6 ∆19–40, Zt6 H70A and Zt6 ∆19–40 H70A proteins by *in vitro* transcription and cell‐free translation. Reactions were incubated for (a, b) 90 min and (c, d) 10–60 min before RNA recovery. No RNA template and luciferase RNA template reactions were used as negative controls.

Cytotoxic RNases, classed as ribotoxins, are known to exert cytotoxicity by disruption of rRNA–protein complexes or cleavage of rRNA with varying degrees of specificity. Despite numerous attempts, we were unable to successfully express FL recombinant Zt6 in *E. coli* or *P. pastoris* protein expression systems. We therefore utilized *in vitro* transcription coupled with a cell‐free protein expression system to assess ribotoxin activity of mature Zt6 and its mutant versions ∆19–40, H70A and ∆19–40 + H70A towards native, functional ribosomes isolated from animal and plant cells (Fig. 3). Full‐length Zt6 showed a ribotoxin‐like activity, cleaving both rabbit (Fig. 3a) and wheat (Fig. 3b) rRNA in an apparent semi‐specific manner, yielding distinct cleavage fragments from both substrates, with greater degradation apparent from rabbit rRNA relative to wheat rRNA. However, although Zt6 H70A and Zt6 ∆19–40 + H70A displayed no catalytic activity against either substrate, Zt6 ∆19–40 activity was indistinguishable from that of wild‐type Zt6 (Fig. 3a,b). Therefore, by contrast with the bona‐fide ribotoxins, the N‐terminal loop appears to be dispensable for Zt6 RNase activity towards native rRNA. To further assess the catalytic activity of Zt6 FL and Zt6 ∆19–40, assays were conducted against both rabbit and wheat rRNA with shortened reaction times (Fig. 3c,d). These experiments indicated that, against rabbit rRNA, reactions containing either Zt6 FL or Zt6 ∆19–40 progressed to completion within 10 min. Longer incubation did not result in further rRNA degradation (Fig. 3c). By contrast, degradation of wheat rRNA was slower for both Zt6 FL and Zt6 ∆19–40 (Fig. 3d), with reactions progressing to completion at between 30 and 60 min of incubation. The fragments produced from the degradation of wheat rRNA by Zt6 FL and Zt6 ∆19–40 were examined by Bioanalyzer (Fig. S1). This analysis confirmed that, similar to ribotoxins, both Zt6 FL and Zt6 ∆19–40 primarily degrade (or attack) the 28S rRNA subunit in a cell‐free expression system.

To further confirm this RNase activity, we used a *P. pastoris* expression system to produce and purify a small quantity of Zt6 ∆19–40 protein. This was tested for RNase activity against both rabbit and wheat ribosomes, alongside the highly specific ribotoxic RNase restrictocin from *Aspergillus restrictus* and the non‐specific RNase A from *Bos taurus* (Fig. S2). These experiments revealed that purified Zt6 ∆19–40 had an activity similar to that of Zt6 ∆19–40 produced in the cell‐free system. The activity of Zt6 ∆19–40 was dissimilar to both the activity of restrictocin, which specifically generates the α‐fragment from 28S rRNA, and RNase A, which non‐specifically degrades all RNA (Fig. S2). Zt6 ∆19–40 displayed non‐specific RNase activity against denatured wheat total RNA, similar to RNase A (Fig. S3). The results indicate that ribosomal structure confers specificity for Zt6 cleavage activity.

### The cytotoxic activity of Zt6 is conserved in the non‐host model plant *N. benthamiana*

**Figure 4:**
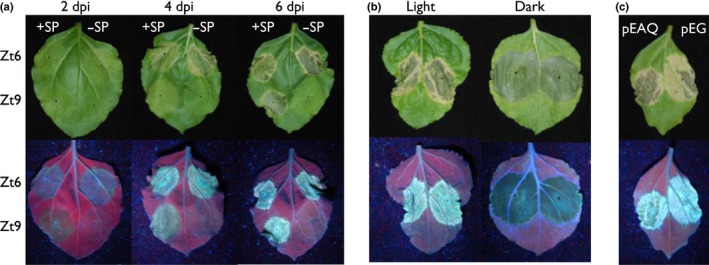
Zt6 is a potent inducer of cell death in the non‐host plant *Nicotiana benthamiana*. (a) Time course of Zt6‐induced cell death in comparison with *Zymoseptoria tritici* effector Zt9 (Kettles *et al*., 2017) following agroexpression. Leaves were photographed at 2, 4 and 6 d post‐inoculation (dpi) and both genes were expressed +/− their native secretion signal peptides (SP). (b) Zt6 (+ SP) agroexpressed in *N. benthamiana* leaves which were subsequently kept under a 16 h : 8 h, day : night cycle (Light) or in 24 h darkness (Dark) for 6 d. Leaves from the same plant are shown. (c) Zt6 (+ SP) agroexpressed from either the pEAQ‐HT‐DEST3 (pEAQ) or pEARLEYGATE101 (pEG) vector and leaves assessed at 7 dpi.

To assess whether Zt6 activity extended beyond the host range of *Z. tritici*, we used *Agrobacterium*‐mediated transient expression (agroexpression) to express Zt6 FL and mutant forms in the non‐host tobacco (*N. benthamiana*) (Fig. 4). These experiments revealed that Zt6 is a highly potent inducer of cell death in this plant species with Zt6‐induced symptoms first visible as early as 2 dpi, which progressed to complete necrosis by 4 dpi (Fig. 4a). In comparison, symptoms induced by other previously characterized *Z. tritici* cell death‐inducing effectors, for example Zt9 (Kettles *et al*., 2017), did not develop until 4 dpi, with widespread cell death in the infiltrated zone not clearly pronounced until 6 dpi (Fig. 4a). Furthermore, Zt6 induced rapid cell death irrespective of whether it was directed to the cytosol or the apoplast, i.e. in the absence or presence of its native SP, respectively (Fig. 4a). By contrast, the previously described Zt9 effector induced cell death only when directed to the apoplast, thus indicating that the mechanism by which Zt6 induces cell death is distinct. Indeed, by contrast with several other previously characterized *Z. tritici* effectors (Kettles *et al*., 2017), which induced light‐ and expression level‐dependent cell death, Zt6 induced cell death in a light‐ and expression level‐independent manner (Fig. 4b,c). Several *Z. tritici* effectors are also known to induce *N. benthamiana* cell death dependent on the Brassinosteroid Insensitive 1 (BRI1)‐Associated Receptor Kinase 1 (BAK1) and Suppressor of BIR1‐1 (SOBIR1) receptor‐like kinases (RLKs). By contrast, we found that Zt6 induced cell death independently of these two RLKs (Fig. S4).

### Zt6 is toxic to prokaryotes and yeasts, but not to *Z. tritici*

**Figure 5:**
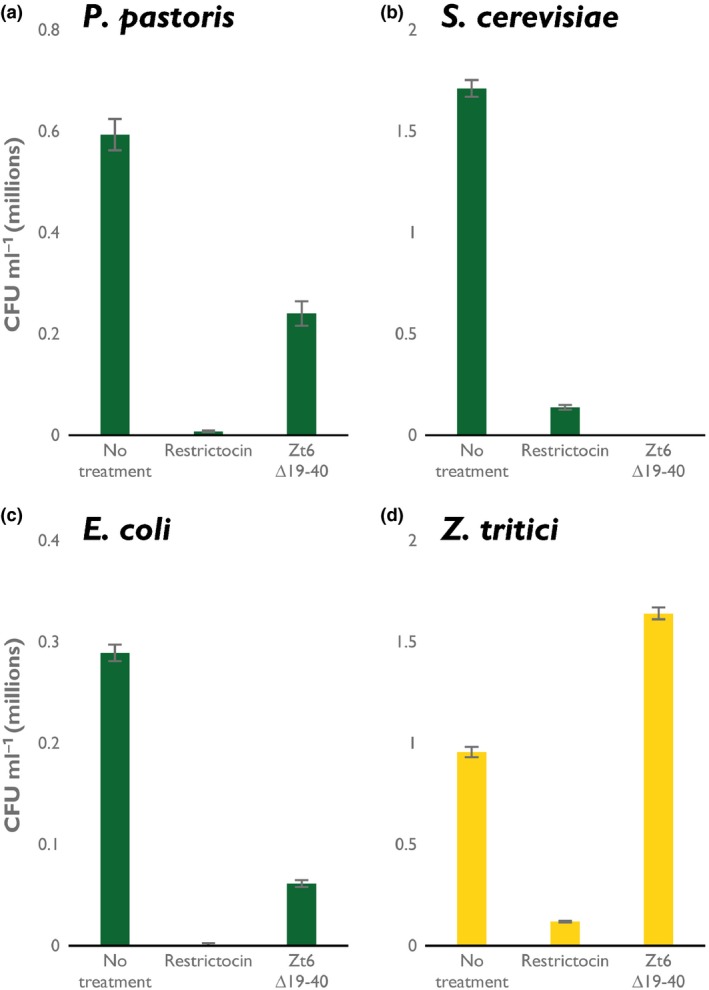
Zt6 is toxic to bacteria and yeasts, but not to *Zymoseptoria tritici*. Recombinant Zt6 ∆19–40 and the ribotoxin restrictocin incubated with (a) *Pichia pastoris*, (b) *Saccharomyces cerevisiae*, (c) *Escherichia coli* and (d) *Z. tritici* at 20 μM concentration. Bars represent mean ± SE.

To assess the contribution of Zt6 to *Z. tritici* virulence on wheat, we created targeted gene deletion mutants using *Agrobacterium*‐mediated transformation. When tested in a wheat infection bioassay, three independent ∆*zt6* transformants displayed similar virulence to the background control strain (∆*ku70*) on wheat cv Riband (Fig. S5). This suggests that Zt6 is not required for wheat infection, although we cannot exclude the possibility of functional redundancy shared with other *Z. tritici* secreted proteins. That Zt6 may be dispensable for wheat infection, coupled with the unusual double‐peak expression pattern during *in planta* infection (Fig. 1a,b) and the potent cytotoxicity against plant cells (Figs 2, 4), led us to speculate that Zt6 may play an alternative role in microbial competition and niche protection. That Zt6 might also exhibit broad antimicrobial toxicity was initially indicated by our failure to produce full‐length recombinant protein in all tested strains of *E. coli* and *P. pastoris*. All colonies recovered as transformants from either system failed to produce protein, and resequencing revealed that they contained mutated, inactivated copies of Zt6 (data not shown). However, we were able to produce small amounts of the Zt6 ∆19–40 mutant in *P. pastoris*. This protein was therefore used in *in vitro* microbial toxicity assays alongside the cytotoxic ribotoxin restrictocin. Restrictocin exhibited potent toxicity against all microorganisms tested: the prokaryote *E. coli*, the yeasts *S. cerevisiae* and *P. pastoris*, and the filamentous fungus *Z. tritici* (Fig. 5a–d). By contrast, Zt6 ∆19–40 exhibited toxic activity against *E. coli*,* S. cerevisiae* and *P. pastoris*, but had no activity against *Z. tritici*. Collectively, these results indicate that Zt6 is cytotoxic against bacteria and the lower yeasts, but not towards *Z. tritici* itself.

## Discussion

Here, we describe the characterization of Zt6, a small sRNase with a ribotoxin‐like activity from the wheat pathogen *Z. tritici* which exhibits potent cytotoxicity towards wheat, tobacco, bacterial and yeast cells. No other secreted protein from this pathogen has been demonstrated to date to have such broad‐spectrum cytotoxicity. The MgNLP effector from *Z. tritici* induces rapid cell death in dicotyledonous plants (*A. thaliana*,* N. benthamiana*), but does not have the same effect in wheat (Motteram *et al*., 2009; Kettles *et al*., 2017). In addition, the secreted proteins ZtNIP1 and ZtNIP2 have been reported to induce cultivar‐specific necrosis/chlorosis in wheat (M'Barek *et al*., 2015). We have recently described a set of *Z. tritici* effectors that induce cell death in *N. benthamiana* (Kettles *et al*., 2017). However, Zt6 has a cytotoxicity spectrum that goes beyond that demonstrated against plant cells in these previous studies to include yeasts and bacteria.

In our recent work, we demonstrated the cell death‐inducing activity of 14 *Z. tritici* secreted proteins in the non‐host plant *N. benthamiana*. Cell death induction by these effectors appeared to be a result of an active recognition in the apoplast by cell surface immune receptors, as it was shown to be light dependent and required the BAK1 and SOBIR1 RLKs. By contrast with those data, we show here that Zt6‐mediated cell death is both light and BAK1/SOBIR1 independent (Fig. S4) and that Zt6 can induce cell death when directed either to the apoplast or the cytosol in both the non‐host *N. benthamiana* (Fig. 4a,d,e) and the natural host plant wheat (Fig. 2b–e). This distinguishes the functionality of Zt6 from that of all previously described effector candidates from *Z. tritici*.

The *Z. tritici* ∆*zt6* mutants displayed no loss of virulence during glasshouse infection assays on the susceptible wheat cultivar Riband (Fig. S5). A similar observation was made during infection of other susceptible wheat cultivars with the same strains (data not shown). This was surprising given the cytotoxic potency demonstrated for Zt6. Given that Zt6 is the only RNase identified in the *Z. tritici* secretome, it seems unlikely that functional redundancy with another secreted RNase can account for the absence of a loss‐of‐virulence phenotype. However, it is possible that other fungal proteins may also contribute to cell death induction, perhaps using a distinct mechanism. However, from the data provided, it is equally likely that the primary function of Zt6 may be to exert cytotoxic activity towards other microorganisms rather than host plant cells during natural *Z. tritici* infections.

Several studies (Mirzadi Gohari *et al*., 2015; Rudd *et al*., 2015; Palma‐Guerrero *et al*., 2016, 2017) have described numerous putative *Z. tritici* effectors based on increased expression *in planta*. Particular focus has been made on those effectors with peak expression shortly before or during the fungal transition to necrotrophy. By contrast, Zt6 displays a rapid induction on spore germination on the leaf surface at 1 dpi (Fig. 1a,b). This induction is more rapid and stronger than for several other previously characterized effectors (Fig. 1b). Given the high level of expression, the phase of early fungal growth on the leaf surface would represent the most likely time for Zt6‐induced host cell death to occur. As it does not (Kema *et al*., 1996; G. Kettles, pers. obs.), it suggests that the Zt6 protein is unlikely to penetrate the waxy leaf cuticle to gain access to the exterior of leaf epidermal cells. This distinguishes Zt6 from, for example, the SnTox1 effector protein from *P. nodorum*, which has recently been shown to be capable of direct penetration of the wheat leaf cuticle to elicit cell death in sensitive cultivars (Liu *et al*., 2016). Therefore, at this point, we hypothesize that the function of Zt6 may be to clear the immediate area around the germinating spore of other potentially harmful or competing microorganisms. By contrast, the period of post‐penetration asymptomatic colonization represents the period of most likely Zt6 entry into leaf mesophyll cells. Given the reduction in Zt6 expression during this phase (4–9 dpi) (Fig. 1a), it suggests that Zt6 activity might be detrimental to fungal colonization were it to induce significant host cell death during what is normally the asymptomatic phase of infection. Finally, the second peak of expression observed during the necrotrophic phase (14–21 dpi) (Fig. 1a) might be interpreted as the niche protective role of Zt6, minimizing colonization of the nutrient‐rich necrotic tissue by opportunistic saprophytic microorganisms.

Structural predictions suggest that Zt6 displays some similarity to the ribotoxin hirsutellin A (Fig. 2a). Ribotoxins have long been known for their potent cytotoxicity and exquisite specificity for cleavage of the phosphodiester backbone at a single nucleotide within the SRL of the 28S rRNA in eukaryotic ribosomes. Although Zt6 also displays considerable potency against numerous eukaryotic cells (Figs 2, 4, 5), the mode of action appears to be different as Zt6 does not generate the characteristic α‐fragment diagnostic of ribotoxin activity (Figs 3, S2, S3). Although this distinction is clear, it is possible that Zt6 may share other features with ribotoxins in order to exert its cytotoxicity. In particular, this may relate to how Zt6 gains access to intracellular RNA when it is initially produced on the exterior of cells. This is exemplified by the requirement of a 22‐amino‐acid N‐terminal loop region for full potency. The classical ribotoxin α‐sarcin also requires N‐terminal amino acids 7–22 for full potency (Garcia‐Ortega *et al*., 2002). The α‐sarcin ∆7–22 mutant retains ribonucleolytic activity against denatured RNA, but is unable to interact with intact ribosomes to generate the α‐fragment. In addition, the ∆7–22 mutant is less efficient at interacting with cell membranes, thus impairing uptake by endocytosis (Garcia‐Ortega *et al*., 2002). It is speculated that other surface‐exposed loops are important for full ribotoxin activity, either through interaction with lipid vesicles or the ribosome itself (Olombrada *et al*., 2017). Given the similarity between Zt6 and α‐sarcin in terms of the requirement for a short N‐terminal region for full potency, these other regions may also function in the ability of Zt6 to both gain access to cells and to exert ribonucleolytic activity.

Given the broad‐spectrum toxicity of Zt6 against plant, yeast and bacterial cells, it is logical to assume that there must exist some mechanism of self‐protection in *Z. tritici*. In bacteria, there exist examples of bacteriocins displaying RNase activity (Riley & Wertz, 2002). Bacterial toxin–antitoxin systems are often based on toxic RNases, immunity to which is often conferred by the presence of dedicated immunity genes carried on the same plasmid (Cook *et al*., 2013). The loss of membrane‐localized susceptibility determinants in the producing cell can also confer RNase immunity (Riley & Wertz, 2002). Mammalian cells secrete RNases with antimicrobial function (Harder & Schröder, 2002; Pulido *et al*., 2013) and are protected themselves by the well‐characterized RNase Inhibitor (RI) protein (Dickson *et al*., 2005). Human cells deficient in RI are themselves more vulnerable to their own sRNases (Thomas *et al*., 2016). It is therefore plausible that an analogous system exists to protect *Z. tritici*. However, the specific inhibitor involved is unknown, as homologues of RI do not exist in the genomes of filamentous fungi. In addition, it is feasible that fundamental differences in lipid composition or physical structure between *Z. tritici* cell membranes and those of bacterial, yeast and plant cells do not permit Zt6 transit. Mechanisms of active efflux that confer xenobiotic tolerance may also offer a measure of self‐protection to any toxic RNase that is able to re‐enter the producing cell. However, at this point, it remains completely unknown how filamentous fungi protect themselves against their own cytotoxic RNases, and this represents a fertile area for future study.

## Author contributions

J.J.R. and K.K. initially conceived the project. G.J.K., J.J.R. and K.K. designed the experiments. G.J.K., C.B., C.A.S. and G.C. conducted all experimental work, and G.J.K. analysed the experimental data. G.J.K., C.A.S., J.J.R. and K.K. wrote the manuscript.

## Supporting information

Please note: Wiley Blackwell are not responsible for the content or functionality of any Supporting Information supplied by the authors. Any queries (other than missing material) should be directed to the *New Phytologist* Central Office.

**Fig. S1** Bioanalyzer separation of wheat germ extract rRNA cleaved by Zt6.
**Fig. S2** Recombinant Zt6 ∆19–40 produced from *Pichia pastoris* is a functional ribonuclease (RNase).
**Fig. S3** Recombinant Zt6 ∆19–40 produced from *Pichia pastoris* degrades denatured RNA non‐specifically.
**Fig. S4** Zt6‐induced cell death in *Nicotiana benthamiana* is independent of the receptor‐like kinases (RLKs) NbBAK1 and NbSOBIR1.
**Fig. S5** The *Zymoseptoria tritici ∆Zt6* mutant is fully virulent on wheat.

**Table S1** Primer sequences used in this study
**Table S2** Accession numbers of the 20 closest Zt6 orthologues within the Dothideomycetes
**Table S3** Pairwise statistics of ‘Cuffdiff’ differential expression analysis for Zt6 across the course of plant infection
