# Supplementary material for: Characterization of an antimicrobial and phytotoxic ribonuclease secreted by the fungal wheat pathogen Zymoseptoria tritici
Source: New Phytol. 2017 Sep 12;217(1):320–31. doi: 10.1111/nph.14786 (PMC5724701; doi:10.1111/nph.14786)
Supplement: Supplementary file 1 — Fig. S1 Bioanalyzer separation of wheat germ extract rRNA cleaved by Zt6. Fig. S2 Recombinant Zt6 ∆19–40 produced from Pichia pastoris is a functional ribonuclease (RNase). Fig. S3 Recombinant Zt6 ∆19–40 produced from Pichia pastoris degrades denatured RNA non‐specifically. Fig. S4 Zt6‐induced cell death in Nicotiana benthamiana is independent of the receptor‐like kinases (RLKs) NbBAK1 and NbSOBIR1. Fig. S5 The Zymoseptoria tritici ∆Zt6 mutant is fully virulent on wheat. [file NPH-217-320-s001.pdf]

New Phytologist Supporting Information Figs S1–S5. Article title: Characterisation of an antimicrobial and phytotoxic ribonuclease secreted by the fungal wheat pathogen *Zymoseptoria tritici*

Authors: Graeme J. Kettles, Carlos Bayon, Caroline A. Sparks, Gail Canning, Kostya Kanyuka and Jason J. Rudd. Article acceptance date: 11 August 2017.

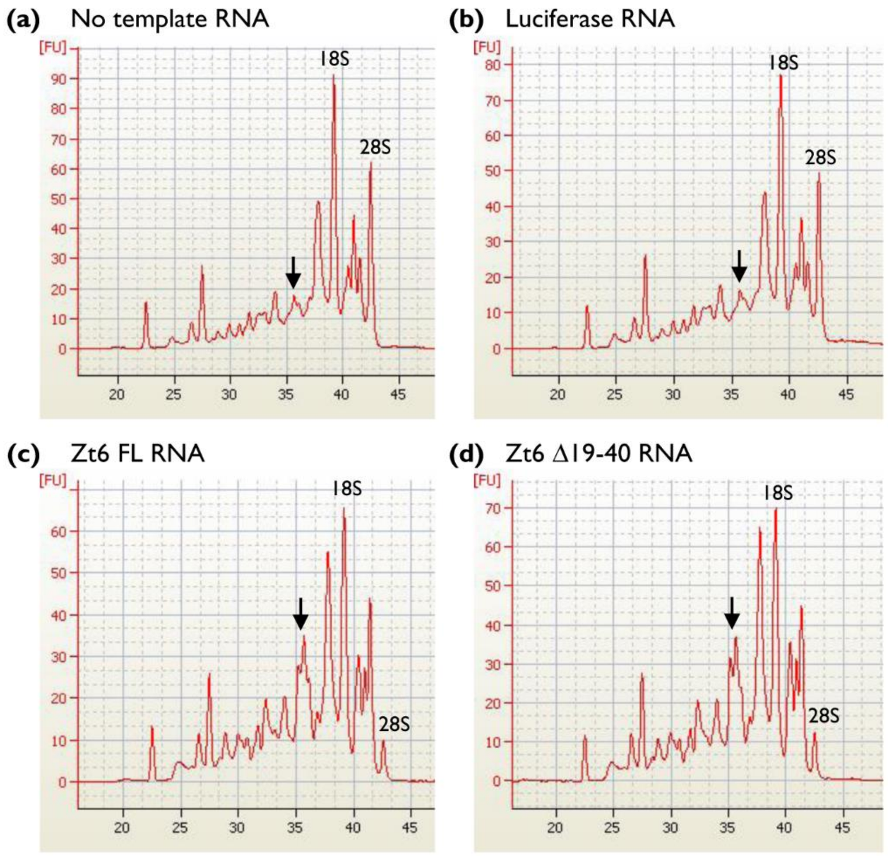

Figure S1. Bioanalyzer separation of wheat germ extract rRNA cleaved by Zt6.

RNA recovered from duplicate reactions to those shown in Fig. 3b. 28S and 18S rRNA peaks indicated. Black arrow indicates cleavage fragments produced by both Zt6 and a Zt6 Δ19-40 mutant, but absent in reactions with either no mRNA template or luciferase mRNA control template.

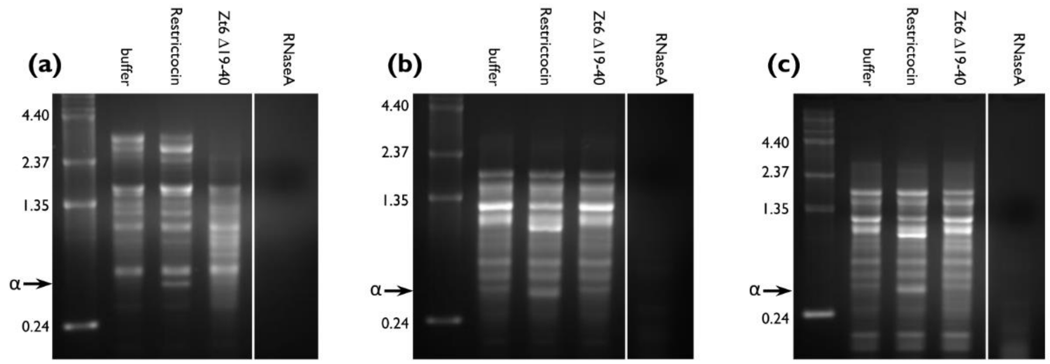

Figure S2. Recombinant Zt6 ∆19-40 produced from *P. pastoris* is a functional RNase.

Zt6 ∆19-40 recombinant protein digests native rRNA from rabbit reticulocyte lysate (a) and wheat germ extract (b, c). Protein concentrations were 85 nM for (a, b) and 850 nM for (c). Arrow indicates production of the α-fragment by restrictocin.

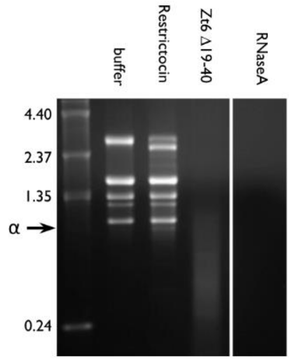

Figure S3. Recombinant Zt6 ∆19-40 produced from *P. pastoris* degrades denatured RNA non-specifically.

Zt6 ∆19-40 protein degrades Trizol-extracted denatured wheat total RNA non-specifically. Proteins used at 85nM. Arrow indicates production of α-fragment by restrictocin.

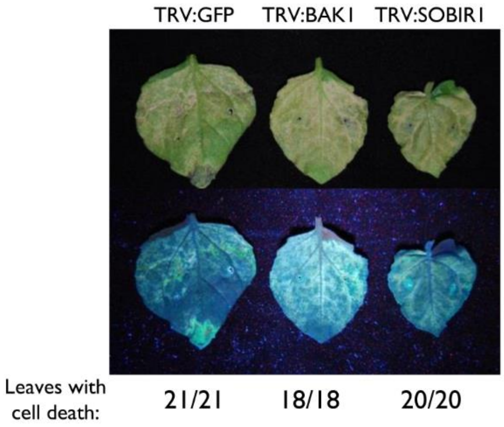

Figure S4. Zt6-induced cell death in *N. benthamiana* is independent of the RLKs NbBAK1 and NbSOBIR1.

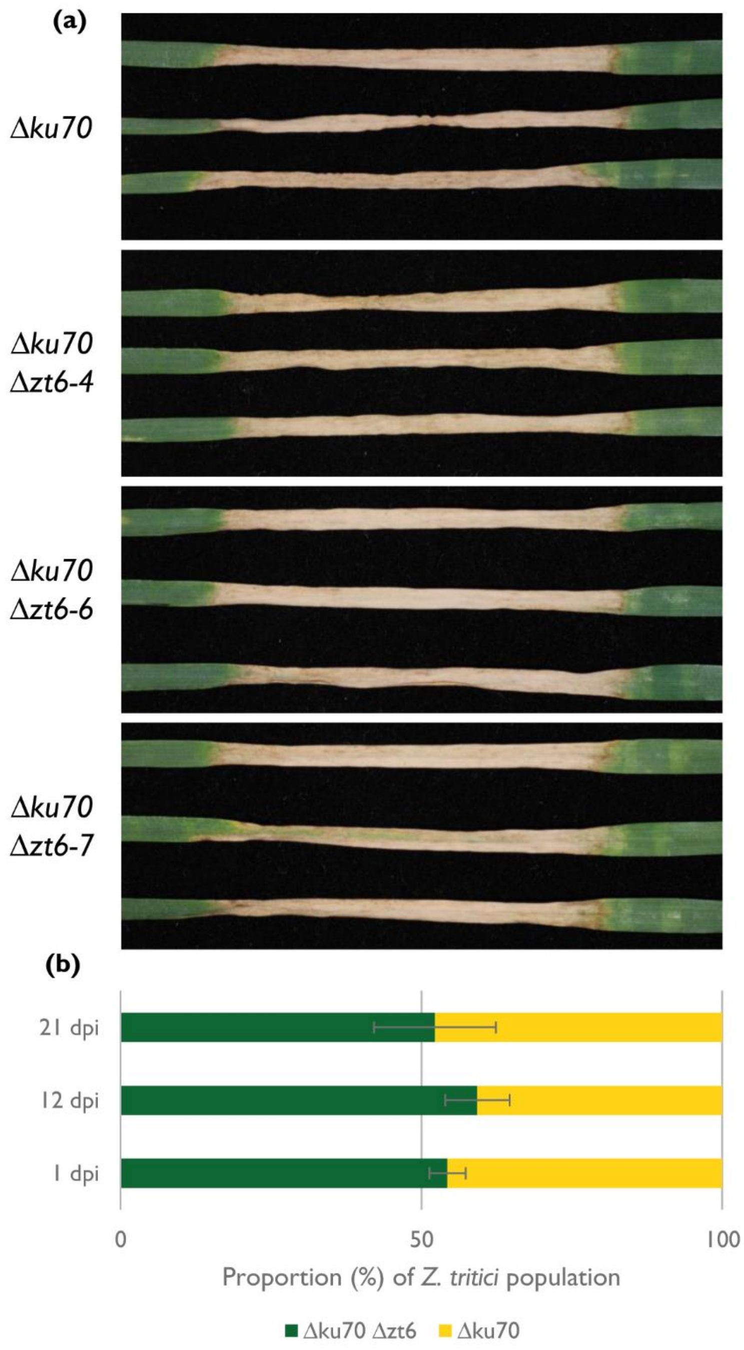

Figure S5. The *Z. tritici* Δ*Zt6* mutant is fully virulent on wheat.

(a) *Z. tritici* Δ*Zt6* bioassay on susceptible wheat cv. Riband. Fungal conidiospores were inoculated onto leaves at 1×10^7 spores/ml and leaves photographed at 18 dpi. (b) Co-inoculation of susceptible wheat cv. Riband with 50:50 mix of *Z. tritici* strains Δ*ku70* (resistant to Geneticin) and Δ*ku70* Δ*zt6* (resistant to Geneticin and Hygromycin) at total inoculum concentration of 1×10^7 spores/ml. Proportion of total *Z. tritici* spore count on leaves assessed by duplicate plating of homogenised leaf samples on YPD supplemented with Geneticin, and YPD supplemented with Geneticin and Hygromycin. Bars represent mean ±SE.
